# Body mass index and cervical cancer screening among women aged 15–69 years in Eswatini: evidence from a population-based survey

**DOI:** 10.1186/s12889-023-16520-y

**Published:** 2023-08-28

**Authors:** Mfundi P. S. Motsa, Wanda Estinfort, Yohane V. A. Phiri, Maswati S. Simelane, Peter A. M. Ntenda

**Affiliations:** 1 Strategic Information Department, Ministry of Health, Mbabane, Eswatini; 2 Behavioral Research and Innovations Unit, Educational Youth Empowerment, Manzini, Eswatini; 3 School of Public Health, College of Public Health, Taipei Medical University, Taipei, Taiwan; 4 Department of Statistics and Demography, Faculty of Social Sciences, University of Eswatini, Kwaluseni, Eswatini; 5 MAC- Communicable Diseases Action Centre (MAC-CDAC), Kamuzu University of Health Sciences (KUHeS), Blantyre, Malawi

**Keywords:** BMI, Cervical cancer, Screening, Residence, Exercise, Eswatini

## Abstract

**Background:**

Cervical cancer stands as one of the most prevalent cancer types among women, despite its preventable nature through early screening and vaccination strategies. The link between being overweight or obese and various adverse health outcomes, including an elevated cancer risk, is well established. Within this study, our central objective was to explore the correlation between body mass index (BMI) and cervical cancer screening (CCS) rates. Moreover, we sought to investigate whether socioeconomic status potentially modulates this relationship.

**Methods:**

Our analysis encompassed 1791 respondents who participated in the World Health Organization’s STEPwise approach to noncommunicable disease risk factor surveillance carried out in Eswatini in 2014. We assessed the connection between BMI, along with other determinants, and CCS through both unadjusted and adjusted logistic regression models.

**Results:**

The uptake of CCS was 14.4% and the prevalence of overweight and obesity was estimated at 28.1 and 34.9% respectively. After accounting for other pertinent variables, the likelihood of obtaining CCS was amplified for individuals classified as obese (adjusted odds ratio [aOR] = 1.99, 95% confidence interval [CI] = 1.26–3.12) or overweight (aOR = 1.98, 95% CI = 1.05–3.74). Furthermore, factors such as being separated or divorced (aOR = 2.03, 95% CI = 1.11–3.72) and engaging in regular physical exercise (aOR = 3.02, 95% CI = 1.21–6.02) were associated with increased odds of undergoing CCS.

**Conclusions:**

This study underscores the noteworthy role played by both overweight and obesity, in conjunction with various socioeconomic factors, in shaping CCS patterns among the surveyed women. For Eswatini, targeted interventions aimed at enhancing CCS participation should take into account the multifaceted factors highlighted within this investigation.

## Background

Cervical cancer stands as one of the most prevalent types of cancer among women. Nevertheless, its preventability through early screening and vaccination strategies has been underscored [1]. The highest number of cases originates from sub-Saharan Africa (SSA), where limited access to cervical cancer screening (CCS) programs and the human papillomavirus (HPV) vaccine has contributed significantly to this burden [2]. Unfortunately, a considerable number of women progress from the early stages of cancer to more advanced ones, and some even face mortality due to the lack of utilization of preventive screening approaches [3]. The SSA region bears a substantial burden of cervical cancer, with roughly 90% of the top twenty affected countries situated in this area. Paradoxically, the adoption of CCS remains alarmingly low (19%) in SSA [4], posing a compelling concern. Notably, in 2018, Eswatini, an area with a high prevalence of HIV, registered the highest incidence rate of cervical cancer [5]. As a part of the Ministry of Health's commitment to cancer prevention and control, a national cancer prevention and control strategy was formulated for the period 2019–2022. This strategy notably places emphasis on elevating the count of facilities providing screening, early detection, and care linkage to 60% [6].

Existing research from the SSA has identified various factors linked to the uptake of CCS. These factors encompass age [7, 8], marital status [9], educational level [10], employment status [11], HIV status [12], smoking [13], alcohol consumption, exercise, susceptibility, and awareness of screening programs [14]. Moreover, there is an emerging interest in investigating the correlation between body mass index (BMI) and CCS in low- and middle-income settings. We posit that a positive association may exist between higher BMI and CCS among women of reproductive age in Eswatini. Several studies have indicated that women with elevated BMI are less inclined to undergo cervical cancer screening compared to those with lower BMI [15, 16]. This might be attributed to factors such as limited healthcare access, challenges in mobility or positioning during the screening procedure, and inadequate knowledge or awareness about the significance of cervical cancer screening [17]. Nonetheless, indirect connections between BMI and cervical cancer screening have been noted [18]. For instance, women with higher BMIs could face an elevated risk of developing cervical cancer due to hormonal imbalances caused by excess body fat, potentially increasing their susceptibility to certain types of cancer, including cervical cancer [19]. Thus, women with high BMI may be cautious to utilize health services such CCS.

A systematic review and meta-analysis focusing on the correlation between BMI and Papanicolaou (Pap) testing in the United States unveiled a positive connection between obesity and CCS among black women [15]. Additionally, studies conducted in Eswatini identified various factors and obstacles related to CCS, including age, educational level [9], prolonged diagnostic procedures [14], misconceptions about cervical cancer causes, and a lack of decision-making authority in health matters [20]. Given Eswatini’s high HIV burden, evidence suggests that HIV infection may alter the impact of cervical cancer's prevalence among women [12]. Despite extensive exploration of risk factors, there remains a gap in the literature concerning BMI and lifestyle factors in the context of Eswatini. The global nutrition report highlighted that 29.2% of adult women (aged 18 years and above) in Eswatini were obese, surpassing the regional average of 20.8% for women [21]. Moreover, a study conducted in rural Eswatini revealed that around 33% of women were overweight and approximately 30% were obese [22]. In light of these considerations, our investigation focused on two primary objectives: first, examining the relationship between BMI, and CCS, and second, exploring the potential moderating role of socioeconomic status (SES) within this association.

## Methods

### Study design and data source

We employed the WHO STEPwise approach to surveillance (STEPS) in a cross-sectional analysis using data from Eswatini's 2014 study. The STEPS methodology was designed to gather information on noncommunicable disease risk factors across the adult population, specifically those aged 15–69 years. Data collection was carried out through face-to-face interviews, employing a multistage cluster sampling technique that identified the sampling frame, as discussed elsewhere. For this analysis, we utilized a sample of 1791 participants within the 15–69 age range. Specifically, our focus was on participants who responded to questions regarding cervical cancer screening in the past 12 months.

### Outcome variable

In this analysis, we focused on the history of CCS within the past year as our main outcome of interest. Participants were given a succinct yet comprehensive explanation of what cervical cancer entails. To gauge this aspect, participants were asked the following question: “Have you ever undergone a screening test for cervical cancer, using any of the methods described above?” Participants who responded with a “yes” to the question were assigned a code of “1,” while those who responded negatively were coded as “0.”

### Main exposure variables

The variables considered as potential influences were body mass index (BMI). Trained data collectors measured anthropometric details, including weight in kilograms and height in meters, using calibrated electronic scales. BMI was derived from weight and height measurements. For analytical purposes, we categorized BMI into different groups: obese (BMI ≥ 30 kg/m2), overweight (BMI ≥ 25 kg/m2), normal weight (BMI ≥ 18.5 and < 25 kg/m2), and underweight (BMI < 18.5 kg/m2), following the classification by [23].

### Behavioral factors

We incorporated three variables to assess the respondents’ behavior: physical exercise (yes/no), smoking habits (yes/no), and alcohol consumption (yes/no). Physical exercise was evaluated through the question, “Do you engage in any activities of moderate intensity, such as sports, fitness routines, or leisurely pursuits like brisk walking, cycling, swimming, and volleyball, for a continuous period of at least 10 min?” Smoking habits were ascertained by inquiring, “Do you presently use any tobacco products, including cigarettes, cigars, or pipes?” Alcohol consumption was determined by asking, “Have you ever partaken in alcohol consumption, such as beer, wine, spirits, or other local variations?”.

### Covariates

We regarded socio-demographic factors, encompassing age, gender, marital status, educational attainment, employment situation, place of residence, and region, as covariates due to their potential influence on our primary variables. The covariates were organized as follows: age groups of respondents (18–30, 31–45, and 46–69 years), marital status categories (married, never married, separated/divorced), educational levels (primary, secondary, high school, and higher education), employment status (employed and unemployed), residential areas (rural and urban), and regions (Hhohho, Manzini, Shiselweni, and Lubombo).

### Statistical* analysis*

We conducted the analysis using Stata version 15 (Stata Corp LP, College Station, TX, USA) from 2017. The analysis was adjusted to account for the complex survey design, which included factors like stratification, non-response, and clustering. Initially, we computed the uptake of CCS among women in Eswatini. Next, we investigated the relationship between BMI and factors associated with CCS, employing the Chi-square test. Subsequently, we employed unadjusted and adjusted models through logistic regression to determine the likelihood of undergoing cervical cancer screening within the past year. Additionally, we explored potential interactions between predictors and sociodemographic variables. The significance threshold for interaction term was set at p < 0.01. In cases where interaction evidence emerged, we conducted a subgroup analysis to explore potential modifying effects. All results from our regression analyses were presented in terms of both crude odds ratios (cORs) and adjusted odds ratios (aORs), accompanied by their corresponding 95% confidence intervals.

### Ethical considerations

We prioritized the principles designed to protect the well-being of human subjects throughout the initial survey process. This commitment led to the approval of the initial STEPS by the Swaziland Ethics Committee. Moreover, we ensured that the custodian of the data was fully informed about our intention to analyze the survey data. To proceed with the data analysis, we obtained explicit permission from the WHO Non-communicable diseases (NCD) Microdata Repository. This repository serves as the responsible entity for managing publicly available data. This comprehensive approach guarantees that ethical considerations were upheld and all necessary approvals were secured before delving into the data analysis process.

## Results

### Distribution of participants by factors associated with past-year suicidal ideation

We conducted an analysis of data from 1791 respondents, revealing that the adoption of CCS stood at 14.4%. The survey participants exhibited predominant traits: obesity (46.3%), an age range of 31 to 45 (40.2%), an educational background spanning high school or beyond (46.3%), and residence in urban settings (62.1%). The characteristics of the respondents linked with CCS are summarized in Table 1, indicating significant correlations (all *p* < 0.01). Notably, factors such as obesity, lack of physical exercise, younger age, marital status (never married), lower educational attainment, unemployment, and urban residency were all strongly associated with CCS (all *p* < 0.01).

**Table 1:** Factors associated with cervical cancer screening

Characteristics	Total N (1791)	Cervical cancer screening		
	*n (%)*	Yes (%, weighted)	No (%, weighted)	*P*-value
BMI				
Underweight	64 (4.3)	4 (1.4)	60 (4.8)	<0.000***
Normal	548 (32.8)	49 (19.1)	499 (35.1)	
Overweight	503 (28.1)	76 (33.1)	427 (27.3)	
Obese	676 (34.9)	127 (46.3)	549 (32.9)	
Exercise				
Yes	240 (15.1)	54 (22.2)	186 (13.9)	0.002**
No	1551 (84.9)	202 (77.8)	1349 (86.1)	
Smoking				
Yes	27 (1.3)	7 (2.4)	20 (1.1)	0.164
No	1764 (98.7)	249 (97.6)	1515 (98.9)	
Alcohol				
Yes	291 (16.4)	54 (21.0)	237 (15.7)	0.127
No	1500 (83.6)	202 (79.0)	1298 (84.4)	
Age				
18–30	646 (49.8)	64 (33.1)	584 (52.6)	<0.000***
31–45	519 (27.8)	102 (40.2)	417 (25.7)	
46–69	626 (22.4)	90 (26.7)	536 (21.7)	
Marital status				
Married	644 (46.1)	76 (32.6)	568 (48.4)	<0.000***
Never Married	865 (42.6)	135 (50.7)	730 (41.3)	
Separated/divorce	282 (11.3)	45 (11.3)	237 (10.3)	
Education level				
Primary and lower	626 (27.2)	53 (18.2)	573(28.7)	<0.000***
Secondary	759 (43.9)	98 (35.5)	661 (45.3)	
High School and higher	406 (28.9)	105 (46.3)	301 (25.9)	
Employment status				
Employed	629 (32.8)	133 (49.1)	496 (30.1)	<0.000***
Not employed	1162 (67.2)	123 (50.9)	1039 (69.9)	
Residence				
Rural	456 (29.5)	96 (37.9)	1175 (71.9)	0.03
Urban	1335 (70.5)	160 (62.1)	360 (28.1)	
Region				
Hhohho	535 (24.6)	74 (24.5)	461 (24.6)	0.61
Manzini	505 (39.6)	89 (43.5)	416 (38.9)	
Shiselweni	333 (15.7)	45 (15.6)	288 (15.7)	
Lubombo	418 (20.1)	48 (16.4)	370 (20.7)	

^*^p value from chi square and Fishers exact tests *p < 0.05; **p < 0.01; *** *p* < 0.005

### BMI and factors associated with CCS

Presented in Table 2 are the outcomes of the univariate, multivariable logistic, and stratified models, employed to examine the connection between BMI and factors associated with CCS. Following adjustments for the other variables listed, certain noteworthy patterns emerged. Notably, individuals classified as obese (aOR = 1.99, 95% CI = 1.26–3.12), as well as those categorized as overweight (aOR = 1.98, 95% CI = 1.05–3.74), demonstrated heightened odds of CCS. Similarly, individuals who were separated or divorced (aOR = 2.03, 95% CI = 1.11–3.72), and those who engaged in regular exercise (aOR = 3.02, 95% CI = 1.21–6.02), exhibited increased odds of experiencing CCS. Conversely, younger respondents (aOR = 0.51, 95% CI = 0.28–0.95), individuals with primary education (aOR = 0.27, 95% CI = 0.14–0.51), and those with secondary education (aOR = 0.38, 95% CI = 0.25–0.58) displayed reduced odds of encountering CCS.

**Table 2:** Predictors and factors associated with cervical cancer screening, stratified by age

Characteristics			Age 15 – 30 years	Age 31–45 years	Age 46–69 years
	cOR (95% CI)	aOR^a^ (95% CI)	aOR^b^ (95% CI)	aOR^b^ (95% CI)	aOR^b^ (95% CI)
Characteristics					
BMI					
Obese	2.58 (1.69 3.92)**	1.99 (1.26 – 3.12)**	2.02 (0.93 – 4.40)	1.47 (1.26 – 3.12)	2.90 (1.18 – 7.12)*
Overweight	2.22 (1.28 – 3.88)**	1.98 (1.05 – 3.74)*	2.17 (0.90 – 5.27)	2.92 (0.93 – 4.40)	2.02 (0.93 – 4.40)
Normal	0.54 (0.16 – 1.84)	0.42 (0.11 – 1.67)	0.26 (0.03 – 2.52)	3.19 (0.93 – 26.3)	2.02 (0.93 – 4.40)
Underweight	1	1	1	1	1
Exercise					
Yes	0.56 (0.36 – 0.90)**	3.02 (1.21 – 6.02)**	1.37 (0.67 – 2.79)	2.93 (1.51 – 5.71)**	2.52 (0.87 – 7.34)
No	1	1	1	1	1
Smoking					
Yes	2.19 (0.70 – 6.82)	2.12 (0.58 – 7.76)	2.04 (0.18 – 22.8)	2.12 (0.58 – 7.76)	3.12 (0.58 – 13.76)
No	1	1	1	1	1
Alcohol					
Yes	1.43 (0.89 – 2.30)	1.13 (0.70 – 1.86)	1.40 (0.55 – 3.57)	1.13 (0.70 – 1.86)	1.13 (0.70 – 1.86)
No	1	1	1	1	1
Age					
18–30	0.51 (0.33 – 0.78)**	0.51 (0.28 – 0.95)*	-	-	-
31–45	1.27 (0.84 – 1.91)	0.98 (0.62 – 1.55)	-	-	-
46–69	1	1	-	-	-
Marital status					
Never Married	1.82 (1.21 – 2.74)**	1.52 (0.96 – 2.41)	1.20 (0.57 – 2.52)	1.52 (0.96 – 2.41)	1.52 (0.96 – 2.41)
Separated/divorce	2.40 (1.48 – 3.89)**	2.03 (1.11 – 3.72)*	2.94 (0.83 – 10.4)	1.18 (0.78 – 3.72)	2.03 (1.11 – 3.72)*
Married	1	1	1	1	1
Education level					
Primary and lower	0.36 (0.22—0.57)**	0.27 (0.14 – 0.51)**	1.11 (0.41 – 2.78)	0.17 (0.07 – 0.41)**	0.27 (0.14 – 0.51)**
Secondary	0.44 (0.30—0.64)**	0.38 (0.25 – 0.58)**	0.76 (0.43 – 1.37)	0.39 (0.19 – 0.76)**	0.38 (0.25 – 0.58)**
High School and higher	1	1	1	1	1
Employment status					
Not employed	0.44 (0.28 – 0.70)	0.79 (0.49 – 1.27)	0.83 (0.40 – 1.73)	0.79 (0.49 – 1.27)	0.79 (0.49 – 1.27)
Employed	1	1	1	1	1
Residence					
Rural	1.56 (1.05 – 2.34)*	1.15 (0.79 – 1.67)	1.32 (0.72 – 2.45)	1.15 (0.79 – 1.67)	1.97 (1.01 – 1.67)*
Urban	1	1	1	1	1
Region					
Hhohho	0.89 (0.57 – 1.40)	0.92 (0.57 – 1.48)	1.09 (0.55 – 2.16)	0.92 (0.57 – 1.48)	0.44 (0.22 – 0.93)*
Shiselweni	0.88 (0.52 – 1.51)	0.87 (0.51 – 1.49)	0.77 (0.32 – 1.85)	0.87 (0.51 – 1.49)	0.87 (0.51 – 1.49)
Lubombo	0.71 (0.43 – 1.17)	0.80 (0.46 – 1.36)	0.98 (0.38 – 2.59)	0.80 (0.46 – 1.36)	0.80 (0.46 – 1.36)
Manzini	1	1	1	1	1

*cOR* crude odds ratio, *aOR* adjusted odds ratio, *CI* Confidence interval

^a^Adjusted for all other listed variables in the model

^b^Adjusted for all other listed variables in the model, excluding age

^*^p < 0.05; **p < 0.01; ***p < 0.005

### Subgroup analysis of BMI and associated factors of CCS stratified different sociodemographic characteristics

Our analysis revealed a potential interaction between educational level and age (*p* < *0.01*), signifying the need for a stratification analysis. Table 3 presents factors linked to cervical cancer screening (CCS), categorized by education level. Respondents engaging in physical exercises demonstrated a higher likelihood of undergoing CCS (aOR = 2.93, 95% CI = 1.51–5.71), unlike their counterparts. Conversely, individuals with primary or lower education (aOR = 0.27, 95% CI = 0.14–0.59) and secondary education (aOR = 0.38, 95% CI = 0.25–0.58) displayed reduced odds of CCS, compared to their peers. Among those aged 46 to 69 years, those classified as obese (aOR = 2.90, 95% CI = 1.18–7.12) and residing in rural areas (aOR = 1.97, 95% CI = 1.01–1.67) exhibited a heightened likelihood of undergoing CCS in contrast to their counterparts. Similarly, individuals within this age group with primary or lower education (aOR = 0.27, 95% CI = 0.14–0.59), secondary education (aOR = 0.38, 95% CI = 0.25–0.58), and hailing from the Hhohho region (aOR = 0.44, 95% CI = 0.22–0.93) displayed reduced odds of CCS. Furthermore, among individuals with high- or higher-level education, a history of exercise (aOR = 2.01, 95% CI = 1.01–3.98) correlated with an increased likelihood of CCS. Conversely, those aged 15 to 30 years (aOR = 0.04, 95% CI = 0.02–0.13), 31 to 45 years (aOR = 0.20, 95% CI = 0.09–0.47), and from the Lubombo region (aOR = 0.16, 95% CI = 0.05–0.51) exhibited diminished odds of undergoing CCS when compared to their counterparts.

**Table 3:** Factors associated with cervical cancer screening, stratified by education level

Characteristics	Primary and Lower	Secondary	High School and Higher
	aOR^a^ (95% CI)	aOR^a^ (95% CI)	aOR^a^ (95% CI)
Characteristics			
BMI			
Obese	2.60 (0.29 – 23.5)	1.80 (0.02 – 3.12)	1.71 (0.77 – 3.84)
Overweight	1.78 (0.49 – 6.55)	1.79 (0.93 – 4.40)	2.14 (0.74 – 6.16)
Normal	2.43 (0.84 – 7.00)	0.20 (0.23 – 1.84)	0.34 (0.27 – 4.35)
Underweight	1	1	1
Exercise			
Yes	1.04 (0.38 – 2.90)	1.76 (0.81 – 3.84)	2.01 (1.01 – 3.98)*
No	1	1	1
Smoking			
Yes	2.48 (0.19 – 30.9)	3.10 (0.58 – 7.76)	0.70 (0.14 – 3.46)
No	1	1	1
Alcohol			
Yes	0.40 (0.14 – 1.11)	1.21 (0.50 – 1.86)	1.75 (0.69 – 4.47)
No	1	1	1
Age			
18–30	2.09 (0.98 – 4.49)	0.82 (0.33 – 2.06)	0.04 (0.02 – 0.13)***
31–45	1.26 (0.49 – 2.99)	1.22 (0.25 –2.58)	0.20 (0.09 – 0.47)***
46–69	1	1	1
Marital status			
Never Married	0.94 (0.29 – 3.07)	1.90 (0.96 – 3.41)	0.92 (0.42 – 2.02)
Separated/divorce	3.69 (0.98 – 13.9)	1.60 (0.60 – 4.34)	1.21 (0.42 – 3.50)
Married	1	1	1
Employment status			
Not employed	1.38 (0.71 – 2.69)	0.97 (0.47 – 1.93)	0.56 (0.23 – 1.40)
Employed	1	1	1
Residence			
Rural	1.38 (0.52 – 3.66)	1.63 (0.94 – 2.84)	1.03 (0.57 – 1.89)
Urban	1	1	1
Region			
Hhohho	1.04 (0.41 – 2.61)	1.18 (0.57 – 2.48)	0.72 (0.33 – 1.54)
Shiselweni	0.67 (0.21 – 2.19)	1.46 (0.51 – 3.49)	0.89 (0.39 – 2.01)
Lubombo	1.20 (0.39 – 3.68)	1.80 (0.96 – 3.52)	0.16 (0.05 – 0.51)**
Manzini	1	1	1

*aOR* adjusted odds ratio, *CI* Confidence interval

^a^adjusted for all other listed variables in the model

^*^p < 0.05; **p < 0.01; ***p < 0.005

## Discussion

Our findings augment the existing body of evidence for Sub-Saharan Africa (SSA) and specifically Eswatini, pertaining to BMI and its correlated elements in relation to cervical cancer screening. The observed uptake of cervical cancer screening in Eswatini (14.4%) falls notably below the regional average of 19% for SSA. This discrepancy may be ascribed to variances in cultural norms [24], disparities in the accessibility of services, and the economic challenges faced [25].

Our study uncovered a noteworthy trend where individuals classified as overweight or obese displayed a higher propensity for undergoing CCS. Additionally, even after segmenting the data by age groups, we demonstrated an elevated likelihood of CCS among individuals with obesity. This observation might stem from the perception of obese and overweight individuals as being in a high-risk category, motivating them to prioritize CCS. Our findings align with prior research [26], validating the notion that overweight and obese individuals are more inclined to engage in CCS. Conversely, contrasting studies have suggested that being overweight or obese does not amplify the likelihood of undergoing cervical cancer screening [15, 16]. Nonetheless, alternate researchers have reported that excess weight could heighten the risk of various cancers, including cervical cancer [27, 28], due to potential hormonal fluctuations induced by excess weight [29]. Broadly, the evidence hints at a connection between overweight/obesity and diminished self-reported health [30, 31], potentially driving individuals with poorer health to seek healthcare services more frequently [32]. This could stem from the necessity for ongoing management of chronic health issues, alongside a greater demand for preventive healthcare services aimed at mitigating future health risks [33]. Moreover, the likelihood of obese women getting screened for cervical cancer is shaped by several factors. Visits to healthcare providers for obesity-related issues offer more opportunities for screenings to be suggested [34]. Some obese individuals, driven by their health awareness, prioritize preventive care like cancer screening [35]. Because obesity is linked to higher risks of various health problems, including certain cancers, healthcare providers are more likely to proactively recommend screenings for these individuals [36]. Furthermore, public health campaigns addressing obesity inadvertently increase awareness about cancer prevention and screenings, motivating obese individuals to participate [37].

We successfully demonstrated a positive correlation between engaging in exercise and an elevated probability of undergoing cervical cancer screening. This connection persisted even after segregating the data by educational attainment, specifically for individuals holding a high school education or beyond. Remarkably, the link between physical exercise and increased odds of cervical cancer screening endured across various educational levels, reinforcing our findings. Our results harmonize with the existing body of knowledge that individuals who incorporate regular physical exercise into their routines exhibit a higher inclination to undergo CCS [38]. However, our observations contrast with conflicting evidence that has associated insufficient physical activity with an augmented likelihood of cervical cancer screening [39]. One plausible explanation for our findings is that individuals adopting healthier lifestyles, characterized by regular exercise, are more predisposed to proactively seek preventive measures, such as cancer screening, as part of their wellness regimen [40]. This underscores the vital role of positive lifestyle choices in promoting early detection and prevention of health issues.

Furthermore, our investigation unveiled several additional insights. We identified that being of a younger age, having a separated or divorced marital status, and possessing secondary or lower educational attainment were correlated with a diminished likelihood of undergoing cervical cancer screening. This finding aligns with established research, which suggests that younger individuals tend to perceive themselves as having a lower risk of cervical cancer [41]. Moreover, women with higher levels of education tend to be more health-conscious and proactive in seeking cancer screening services [2]. Interestingly, within the cohort of highly educated women, those hailing from the Lubombo region exhibited a reduced likelihood of cervical cancer screening. Our findings suggest that this discrepancy could potentially be attributed to variations in regional norms and cultural practices across the country. The influence of regional factors on health-seeking behavior underscores the need for tailored strategies that consider local contexts and sensibilities when promoting healthcare services.

Our study exhibits both inherent strengths and limitations. One of our main strengths lies in the utilization of a nationally representative survey, ensuring a robust and diverse sample. With an adequate sample size, our findings possess a level of statistical reliability. The credibility of our data collection tools is bolstered by their prior application in multinational surveys, thereby enhancing the trustworthiness of our results. Moreover, the precision of our BMI measurements, carried out using validated scales, lends further credence to our findings. However, it’s important to approach our results cautiously due to certain limitations. Our study’s design is cross-sectional in nature, which means that while we've identified associations, causality cannot be definitively inferred. Additionally, the secondary analysis nature of our study might influence the extent of associations observed. Furthermore, while our study sheds light on important factors related to CCS, it's imperative to acknowledge that our results present associative possibilities rather than established causal links. To attain a more comprehensive understanding of CCS, future investigations could consider incorporating validated scales like the Health Literacy Questionnaire (HLQ) to assess health literacy and indicators of awareness about HPV vaccination [42]. In summation, our study’s strengths encompass its use of a representative survey and validated measurement tools, while its limitations include the cross-sectional design and the need for cautious interpretation of associations. This study serves as a stepping stone for future research to delve deeper into the intricate dynamics of cervical cancer screening and related factors.

Our findings offer valuable insights that hold the potential to shape policy decisions. Particularly, as the national cancer control and prevention program embarks on revising its strategic plan, our results underscore the necessity of extending focus towards cervical cancer screening, with a specific emphasis on overweight and obese individuals. This demographic emerges as a high-priority group warranting targeted interventions. Moreover, within the high-risk groups identified, a targeted approach to cervical cancer screening is recommended. Specifically, for those with a high school education or beyond, it is prudent to concentrate efforts on screening individuals within the middle and younger age ranges. This targeted approach aligns with our findings and could effectively enhance the effectiveness of cervical cancer screening initiatives. Incorporating these insights into policy decisions could contribute to a more comprehensive and effective national strategy for cervical cancer control and prevention. By prioritizing the high-risk groups we've identified and tailoring screening efforts based on educational levels and age brackets, we can potentially optimize the impact of preventive measures and improve overall cervical cancer screening rates..

## Conclusions and recommendations

Our results revealed significant associations between certain factors and cervical cancer screening. Specifically, being obese, having a secondary or lower level of education, residing in rural areas, and belonging to the Lubombo region were linked to cervical cancer screening behaviors. These findings emphasize the urgency for further in-depth behavioral studies that employ rigorous methodologies. Such studies could delve into the underlying reasons behind the low uptake of cervical cancer screening, offering a more nuanced understanding of this issue. Furthermore, our results underscore the imperative of promoting early engagement in cancer screening services among women. By encouraging early participation, we can facilitate the early detection and subsequent treatment of cervical cancer cases. This not only enhances the chances of successful treatment but also underscores the significance of proactive healthcare-seeking behaviors. In essence, our findings advocate for a two-fold approach: the pursuit of comprehensive behavioral studies to uncover the root causes of low screening rates, and the implementation of initiatives that raise awareness and encourage early involvement in cervical cancer screening, ultimately contributing to improved public health outcomes.

## Data Availability

The datasets used or analyzed were with permission from the WHO NCD Microdata Repository, which serves as custodian of publicly available data; data are available on the website https://extranet.who.int/ncdsmicrodata/index.php/catalog
